# DNA-Based Sensor for the Detection of an Organophosphorus Pesticide: Profenofos

**DOI:** 10.3390/s18072035

**Published:** 2018-06-25

**Authors:** Giulia Selvolini, Ioana Băjan, Oana Hosu, Cecilia Cristea, Robert Săndulescu, Giovanna Marrazza

**Affiliations:** 1 Department of Chemistry “Ugo Schiff”, University of Florence, Via della Lastruccia 3, 50019 Sesto Fiorentino (FI), Italy; giulia.selvolini@unifi.it (G.S.); bajanioana@yahoo.com (I.B.); hosu.oana@umfcluj.ro (O.H.); 2 Department of Analytical Chemistry, University of Medicine and Pharmacy “Iuliu Haţieganu”, 4 Pasteur Street, 400349 Cluj-Napoca, Romania; ccristea@umfcluj.ro (C.C.); rsandulescu@umfcluj.ro (R.S.); 3 Istituto Nazionale Biostrutture e Biosistemi (INBB), Unit of Florence, Viale delle Medaglie d’Oro 305, 00136 Roma, Italy

**Keywords:** screen-printed electrodes, aptasensor, nanoparticles, organophosphorus pesticide, profenofos

## Abstract

In this work, we propose an electrochemical DNA aptasensor for the detection of profenofos, an organophosphorus pesticide, based on a competitive format and disposable graphite screen-printed electrodes (GSPEs). A thiol-tethered DNA capture probe, which results to be complementary to the chosen aptamer sequence, was immobilised on gold nanoparticles/polyaniline composite film-modified electrodes (AuNPs/PANI/GSPE). Different profenofos solutions containing a fixed amount of the biotinylated DNA aptamer were dropped onto the realized aptasensors. The hybridisation reaction was measured using a streptavidin-alkaline phosphatase enzyme conjugate, which catalyses the hydrolysis of 1-naphthyl -phosphate. The 1-naphtol enzymatic product was detected by means of differential pulse voltammetry (DPV). The aptasensor showed itself to work as a signal off sensor, according to the competitive format used. A dose response curve was obtained between 0.10 μM and 10 μM with a detection limit of 0.27 μM.

## 1. Introduction

“Pesticide” is a term used in a broad sense to address any substance or mixture of substances used to limit the growth of infesting species (e.g., insects, weeds, little mammals, fungi, etc.) that can compromise agricultural production. As stated by the Food and Agriculture Organization (FAO), a pesticide is defined as substances intended to kill or control pests, but, for the present purposes, it also embraces certain materials used to modify the behaviour or physiology of pests (e.g., insect repellents and synergists) or of crops during production or storage (herbicide safeners, germination inhibitors) [1]. Pesticide residues may gain access to the food chain through air, water and soil, which is an outstanding issue to face, since most of the substances used as pesticides are constituted of neurotoxic compounds.

Organophosphorus pesticides (OPs) like phorate, profenofos, and isocarbophos are highly toxic substances that irreversibly inhibit the enzyme acetylcholinesterase, an essential enzyme for the central nervous system to function properly both in humans and insects. This inhibition causes the accumulation of the acetylcholine neurotransmitter in nerves, which affects muscular activities and the normal functioning of vital organs, causing severe symptoms and even death [2,3]. Thus, the analysis of pesticide residues is an important concern due to their bioaccumulation effect, high toxicity, and long-term damage risk to environment and human health security.

In 2013, the Acceptable Daily Intake (ADI) was established for profenofos as 0–0.03 mg/kg body weight from the Codex committee, FAO, and WHO. The highest level of a pesticide residue that is legally tolerated in or on food depends on its nature.

To date, the detection and the quantification of the pesticides are generally based on conventional chromatographic techniques coupled with mass spectroscopy [4], which provide sensitive and selective detection. In particular, profenofos have been carried out both through a gas–liquid chromatographic method using a flame-ionization detector [5] and an HPLC-based method using a diode array detector (DAD) [6].

The chromatographic techniques commonly require highly skilled personnel and are not suitable for screening analysis. Thus, biosensor development for pesticide analysis represents a rapid, cost-effective, and easy alternative to conventional techniques for environmental monitoring, including in situ analysis [7]. The main drawback in developing biosensors for detecting pesticides consists in the synthesis of antibodies for these highly toxic targets. In this perspective, the use of bio-mimetic receptors such as DNA aptamers has recently become an interesting alternative, since they have shown themselves as good candidates as recognition elements in more robust and stable biosensors for pesticide detection [8,9,10].

Recently, some aptamer-based biosensors (aptasensor) have been developed for the determination of OPs. A colorimetric assay was developed employing gold nanoparticles (AuNPs) modified with aptamer for the detection of omethoate. The aptamer showed high selectivity towards omethoate, resulting in the disconnection of aptamer molecules from AuNPs and in their aggregation. Using the OP-binding aptamer and target-induced colour changes in AuNPs, this biosensor showed a good linearity between 0.1 μM and 10 μM, with LOD of 0.1 μM [11]. A similar approach was developed for the colorimetric detection of malathion employing aptamer, cationic peptide and unmodified gold nanoparticles. The biosensor was found to be linear in the range of 0.01–0.75 nM with a limit of detection of 1.94 pM [12].

The use of electrochemical platform for aptasensors leads to many advantages such as realization of arrays for multidetections, device miniaturization, and user-friendly and low-cost apparatus.

An electrochemical aptasensor based on copper oxide nanoflowers and single-walled carbon nanotubes nanocomposite for chlorpyrifos detection was developed by Huo et al. A good linearity for chlorpyrifos ranging from 0.1 ng/mL to 150 ng/mL, with a low detection limit of 70 pg/mL, was obtained [13].

Different optical and photoelectrochemical affinity sensors for profenofos detection, based on molecularly imprinted polymers (MIPs) and calixarenes, have been reported in the literature [14,15,16].

In this work, we developed, for the first time, an electrochemical DNA aptasensor for profenofos based on a competitive assay format using screen-printed cells (SPCs). SPCs are widely used as electrochemical platform for aptasensors for various advantages such as the simple, rapid, and inexpensive manufacturing process. In addition, their low cost permits the possibility of single use in order to avoid the contamination among the samples.

The DNA aptamer was selected from a library of aptamers, built by SELEX, that proved itself to show one of the highest ability to bind profenofos among other organophosphorus pesticides [17]. A thiol-tethered DNA capture probe, which is complementary to the chosen aptamer sequence, was immobilised on gold nanoparticles/polyaniline composite film (AuNPs/PANI) modified graphite screen-printed electrode surface. Different profenofos solutions containing a fixed amount of the biotinylated DNA aptamer were dropped onto the realised aptasensors. The hybridisation reaction was measured using a streptavidin-alkaline phosphatase enzyme conjugate, which catalyses the hydrolysis of 1-naphthyl-phosphate to 1-naphthol. The enzymatic product was detected by means of differential pulse voltammetry (DPV). The aptasensor showed itself to work as a signal off sensor, according to the competitive format used. Finally, preliminary experiments were carried out with fruit juices.

This innovative method combines the portability of screen-printed electrochemical cells and of a computer-controlled instrument to ensure the possibility of a disposable and cost-effective in situ analysis.

## 2. Materials and Methods

### 2.1. Chemicals

Aniline, perchloric acid (HClO_4_), tetrachloroauric acid (HAuCl_4_), sulfuric acid (H_2_SO_4_), potassium ferrocyanide (K_4_[Fe(CN)_6_]), potassium ferricyanide (K_3_[Fe(CN)_6_]), DL-dithiothreitol (DTT), 6-mercapto-1-hexanol (MCH), di-sodium hydrogen phosphate (Na_2_HPO_4_), sodium di-hydrogen phosphate di-hydrate (NaH_2_PO_4_·2H_2_O), profenofos, paraoxon, di-ethanolamine (DEA), potassium chloride (KCl), magnesium chloride (MgCl_2_), bovine serum albumin (BSA), streptavidin-alkaline phosphatase enzyme conjugate, 1-naphthyl-phosphate, and *p*-nitrophenyl phosphate were purchased from Sigma-Aldrich (Milan, Italy) at analytical grade. Milli-Q water was used for all preparations.

The DNA sequences were purchased from Eurofins Genomics (Ebersberg, Germany) and are listed below:

- thiol-tethered oligonucleotide complementary sequence (oligo-SH): 5′-(SH)-(CH_2_)_6_-CCG ATC AAG AAT CGC TGC AG-3′;
- biotinylated DNA aptamer (apt-BIO): 5′-(biotin)-TEG(triethylene glycol)-AAG CTT GCT TTA TAG CCT GCA GCG ATT CTT GAT CGG AAA AGG CTG AGA GCT ACG C-3′.

Prior to immobilisation, the thiol-modified DNA sequence was treated with dithiothreitol (DTT), purified by elution through NAP-5 columns of Sephadex G-25 DNA grade resin (GE Healthcare, Amersham Place, UK) and then quantified by measuring UV absorption at 260 nm.

The buffer solutions used in this work are

1. storage buffer: 10.0 mM TRIS buffer, pH 8.0;
2. immobilisation buffer: 0.50 M phosphate buffer, pH 7.0;
3. detection buffer: 0.10 M DEA buffer, 0.10 M KCl, 1.0 mM MgCl_2_, pH 9.6.

### 2.2. Apparatus

UV absorption measurements were carried out through a Varian Cary 100 Bio UV-spectrophotometer equipped with a 6 + 6 peltier thermostatable multicell holder and built-in temperature probes. The results were analysed with the Thermal application provided in the Cary 100 Bio software suite (Agilent, Cernusco sul Naviglio Milan, Italy). Secondary structures of both the DNA sequences were predicted through the Mfold algorithm [18].

Electrochemical measurements were carried out with a portable potentiostat/galvanostat PalmSens electrochemical analyser (PalmSensGA Houten, The Netherlands), and the results analysed by PSTrace 2.3 software. All the reported potentials refer to the pseudo-reference silver screen-printed electrode and all the measurements were carried out at room temperature.

The aptasensors were realised using screen-printed cells formed by graphite working electrode (3 mm diameter), a silver pseudo-reference electrode and a graphite counter electrode (GSPEs). The screen-printed cells were purchased from EcoBioServices (Florence, Italy).

### 2.3. DNA Melting Curve Studies

The hybridisation reaction between the DNA capture aptamer (apt-BIO) and the selected complementary sequence (oligo-SH) was assessed by recording melting curves. Melting temperatures were obtained as first-order derivative plot of absorbance versus temperature.

Herein, 100 μL of 1.0 μM oligonucleotide solutions in immobilisation buffer were placed into quartz microcuvettes (1 cm path length), and the temperature was increased from 25 °C to 95 °C at a constant rate of 1.0 °C/min directly inside them through the immersed probe into the sample solutions. At the same time, the absorbance at 260 nm was monitored at 1 nm spectral bandwidth. Immobilisation buffer was used as a blank solution. The entity of the interaction between the DNA aptamer and the target pesticide was also investigated in the same conditions.

### 2.4. Aptasensor Development

#### 2.4.1. GSPEs Surface Modification by Electrodeposition of Polyaniline and Gold Nanoparticles

**Figure 1:**
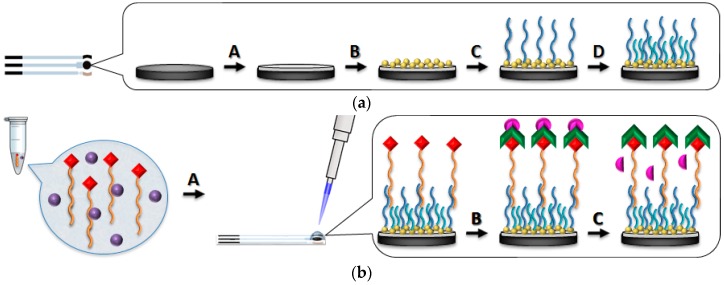
Scheme of the DNA-based sensor assay for profenofos detection. GSPE surface modification (**a**) (A) electropolymerisation of polyaniline using 2.5 mM of aniline solution in 50 mM HClO_4_ by cyclic voltammetry (potential range: −400 mV → +800 mV; scan rate: 50 mV/s; 10 cycles); (B) electrodeposition of gold nanoparticles using 0.5 mM HAuCl_4_ solution in 0.5 M H_2_SO_4_ by cyclic voltammetry (potential range: −200 mV → +1200 mV; scan rate: 100 mV/s; 15 cycles); (C) thiolated DNA capture probe immobilisation from 2.0 μM solution in 0.50 M phosphate buffer, pH 7.0 (incubation time: overnight); (D) mixed SAM formation with 1.0 mM solution of 6-mercapto-1-hexanol (incubation time: 60 min); Hybridisation and detection (**b**) affinity reaction between profenofos samples and 0.5 μM biotinylated aptamer solution (incubation time: 40 min); (A) hybridisation reaction between DNA aptamer and immobilised DNA capture probe (incubation time: 30 min); (B) coupling with streptavidin-alkaline phosphatase enzyme conjugate (1.0 U/mL in 0.10 M DEA buffer, 0.10 M KCl, 1 mM MgCl_2_, pH 9.6; incubation time: 10 min); (C) incubation with 1.0 mg/mL 1-naphthyl phosphate solution in DEA buffer for 20 min and electrochemical detection of 1-naphthol by DPV (potential range: 0 mV → +600 mV; scan rate: 40 mV/s; step potential: 5 mV; modulation amplitude: 70 mV).

The developed DNA aptasensor assay was based on a competitive approach, as reported in Figure 1.

Polyaniline (PANI) and gold nanoparticles (AuNPs) modified GSPEs were realised in accordance with the optimised procedure reported in our previous works [10,19].

Briefly, electropolymerisation of aniline was performed through cyclic voltammetry (CV) by dropping 50 μL of 2.5 mM aniline solution in 50 mM HClO_4_ onto GSPEs. The potential was scanned from −400 mV to +800 mV for 10 cycles at 50 mV/s scan rate.

After washing with 50 μL 0.5 M H_2_SO_4_, AuNPs were deposited through CV by dropping 50 μL of 0.5 mM HAuCl_4_ solution in 0.5 M H_2_SO_4_ onto the polymeric layer. The potential was varied from −200 mV to +1200 mV at 100 mV/s for 15 cycles.

The modified GSPEs were then washed three times with 50 μL milli-Q water to remove excess monomer and free ions from the surface.

#### 2.4.2. Electrochemical Characterisation of the Modified GSPEs

Each modification step of the developed platform was electrochemically characterised through CV by dropping 50 μL of 5.0 mM [Fe(CN)_6_]^3−/4−^ redox probe (equimolar solution in 0.1 M KCl) onto the screen-printed cells (SPCs). CV measurements were performed in the potential range from −500 mV to +800 mV at 150 mV/s scan rate. After the electrochemical analysis, the SPCs were discarded.

#### 2.4.3. DNA Probe Immobilisation

The nanostructured GSPEs were modified by self-assembly of a mixed monolayer of thiolated DNA capture probe (oligo-SH) and MCH [20]. The purified thiolated complementary sequence was subjected to a thermal treatment by heating it at 90 °C for 5 min and cooling it down to room temperature.

Then, 7.0 μL of the 2.0 μM capture probe solution were then deposited onto the modified surface of the working electrode and chemisorption was allowed to proceed overnight (≈16 h). During this period, the sensors were stored in petri dishes at 4 °C to protect the solution from evaporation. To remove unbound oligonucleotide sequences, the surface was washed three times with 15 μL of immobilisation buffer.

This immobilisation step was followed by the formation of a self-assembled monolayer (SAM) formation by incubation with 7.0 μL of 1.0 mM MCH aqueous solution for 60 min. Finally, the aptasensors were washed with 15.0 μL immobilisation buffer for three times.

#### 2.4.4. Profenofos Detection

To obtain a dose-response calibration curve, profenofos detection was performed by dropping a solution containing a proper concentration of biotinylated DNA aptamer (apt-BIO) containing the target pesticide onto the sensor surface and by allowing the competitive reaction to proceed.

In particular, the affinity reaction between 0.50 μM biotinylated DNA aptamer and target pesticide in the concentration range 0–10.0 μM was first performed into solution; then, after 40 min, 7.0 μL of these solutions were incubated for other 30 min onto the sensor surface.

The aptasensors were then rinsed for three times with 15.0 μL detection buffer.

#### 2.4.5. Enzymatic Labelling and Electrochemical Measurements

The biotinylated hybrids formed onto the developed aptasensor surface were further incubated with 15.0 μL of a solution containing 1.0 U/mL of streptavidin-alkaline phosphatase conjugate and 8.0 mg/mL of BSA in detection buffer. After 10 min, each sensor was washed three times with 100 μL detection buffer for two cycles of washings.

Then, after this labelling step, 50.0 μL of 1.0 mg/mL 1-naphthyl-phosphate solution in the detection buffer were placed onto the disposable aptasensors. After 20 min, the electroactive enzymatic product thus formed (1-naphthol) was detected by differential pulse voltammetry (DPV) by scanning the potential from 0 mV to 600 mV at 40 mV/s (5 mV step potential, 70 mV modulation amplitude) [21].

The current peak height was taken as the electrochemical signal. The signal is expressed in relative percentage units as S_x_/S_0_ (e.g., ratio between measured signal to blank signal) and plotted versus profenofos concentration. The obtained curve exhibits the typical sigmoidal shape of a competitive assay and was fitted by using OriginPro 8.5 software (OriginLab) with a Boltzmann-type sigmoidal equation [22]:

(1) $$y=A_{2}+\frac{A_{1}-A_{2}}{1+e^{\left(x-x_{0}\right)/dx}}$$

where *A*_1_ is the *y* value at the top plateau at the curve, *A*_2_ is the *y* value at the bottom plateau, *x*_0_ is the *x* value at which *y* is halfway between bottom and top and *dx* is the slope of the linear part of the curve.

## 3. Results and Discussion

### 3.1. Studies on the Affinity of the DNA Aptamer for the Target Pesticide

**Figure 2:**
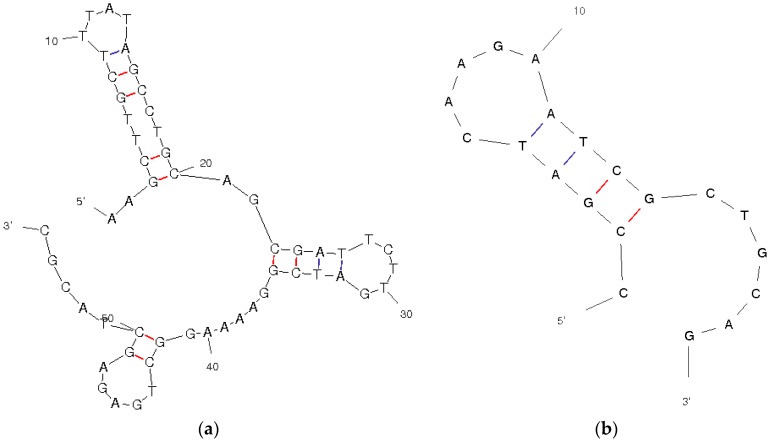
Secondary structures of apt-BIO (**a**) and oligo-SH (**b**) sequences as predicted with the MFold algorithm at 25 °C in 0.50 M phosphate buffer, pH 7.0, and 0.8 M Na^+^ concentration. The bond between G and C is shown in red to indicate its stability.

The aptamer was selected from a library of aptamers, built by SELEX that shows the highest ability to bind profenofos among other organophosphorus pesticides. The constant of dissociation was estimated K_d_ = 1 µM [17]. The selective binding of the analytes is strongly influenced by the secondary structures of the DNA sequences, since aptamers themselves are subjected to conformational changes that create many weak bonds, in order to capture the target molecule. The secondary structures thus formed were predicted by using the MFold algorithm and are shown in Figure 2.

The prediction temperature was set at 25 °C, and the ionic strength was regulated according to the buffer solution used. The drawing mode was set to untangle with loop fix, while the other parameters were left as default settings. From the predicted conformations, apt-BIO prevalently shows a single-stranded structure characterised by three differently sized loops, while oligo-SH shows a single-loop structure in the same conditions because of its shorter length. For this reason, the hybridisation reaction between the two DNA strands will be more efficient compared to the one that would occur in presence of any other sequence with a more stable secondary structure.

The affinity of the receptor for the analyte and for the chosen complementary sequence was assessed by comparing these structures with preliminary studies on the melting temperatures of the aptamer alone and in presence of its complementary sequence or the pesticide (Table 1).

The obtained T_m_ values for the biotinylated aptamer in presence of the thiolated capture probe or profenofos are close to the one related to the aptamer alone; moreover, the overlapping region of the two oligonucleotides is located on the medium-size loop. This suggests that also the interaction with profenofos takes place in the same region.

### 3.2. Aptasensor Development

#### 3.2.1. Modification of GSPEs with Polyaniline and Gold Nanoparticles

The GSPEs were first modified with a layer of the conducting polymer polyaniline. Since conducting polymers show numerous features suitable for their application in sensing and biosensing (e.g., low cost, flexibility and biocompatibility), they have recently attracted a lot of attention in this field.

Here, the polymer is used to provide higher electroactive area (compared to the bare electrode), protection versus fouling of the electrode surface and scaffold for dispersing and anchoring the metal particles. Inclusion of AuNPs in conductive polymers can enhance electron transfer through a direct or mediated mechanism with improved conductivity and enhanced stability [14]. Further, the AuNPs are an excellent substrate for the immobilisation of thiolated bioreceptor.

The polymerisation profile shows that, for potentials values around +0.65 V, the formation of the monomer radical decreases with the number of cycles, probably due to the fact that the polymer formed during the first cycles prevents the monomer from reaching the surface of the electrode. Thus, the growth of chains already formed is mainly occurring.

**Figure 3:**
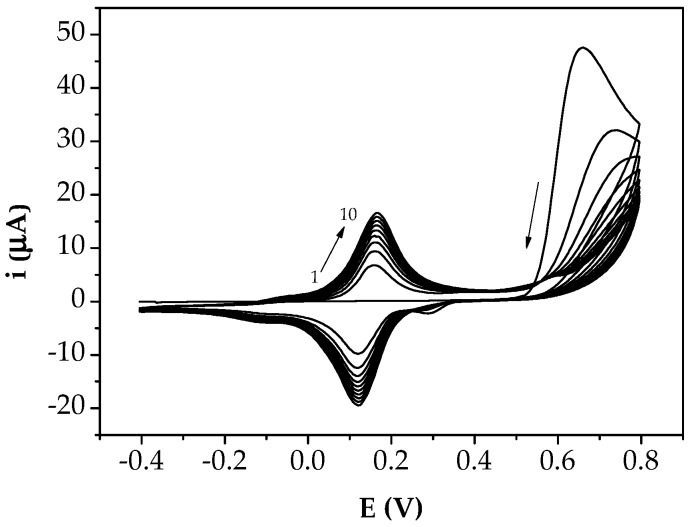
Electropolymerisation of 2.5 mM aniline solution in 50 mM HClO_4_ at GSPE surface by cyclic voltammetry (potential range: −400 mV → +800 mV; scan rate: 50 mV/s; 10 cycles).

These voltammograms still show, at about 0.14 V vs. Ag/AgCl, the redox pair corresponding to the oxidation/reduction of PANI, the peak increasing in height with the number of cycles as the PANI is formed (Figure 3).

**Figure 4:**
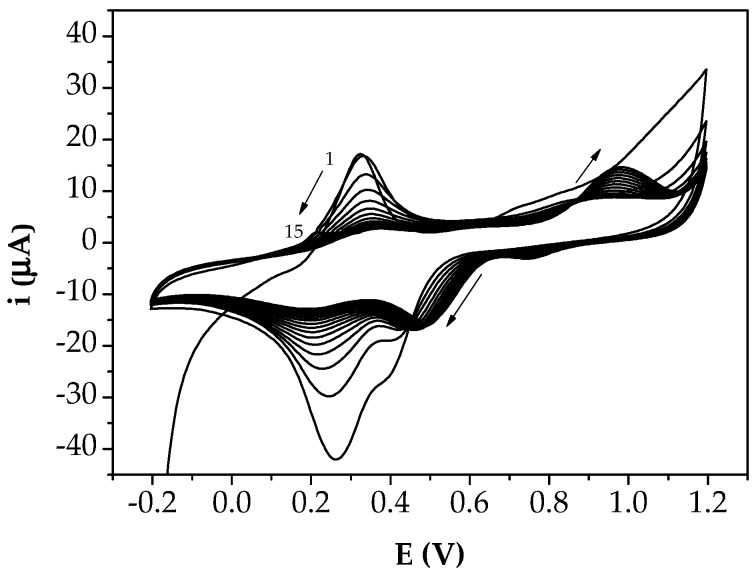
Electrodeposition of gold nanoparticles at PANI/GSPE surface from a 0.5 mM HAuCl_4_ solution in 0.5 M H_2_SO_4_ by cyclic voltammetry (potential range: −200 mV → +1200 mV; scan rate: 100 mV/s; 15 cycles).

The number of cycles of electropolymerisation was chosen according to the data from the cyclic voltammetry, considering the growth of the faradaic current with each cycle. However, the application of 10 cycles to obtain the PANI/GSPEs was more adequate for the desired purpose, since this gave a more stable signal. When AuNPs were electrodeposited on the PANI/GSPEs by cyclic voltammetry, the current peak dramatically increased (Figure 4).

**Figure 5:**
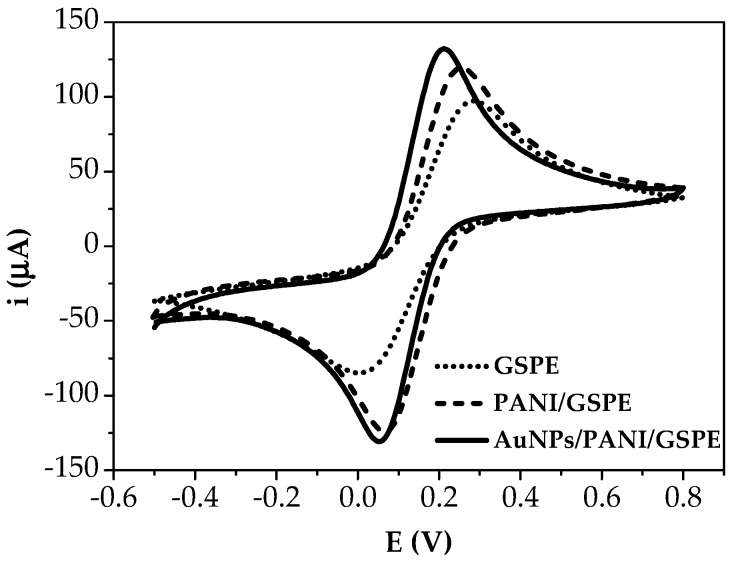
Electrochemical characterisation of modified SPEs by cyclic voltammetry (potential range: −500 mV → + 800 mV; scan rate: 150 mV/s) by using the Fe(CN)_6_^4−/3−^ redox couple (5.0 mM solution in 0.1 KCl).

In particular, the cooperation of PANI and AuNPs in the modified film amplified the peak current and reversibility of redox peaks, which can be related to the larger electroactive surface area of AuNPs/PANI/GSPEs and electrocatalytic behaviour of gold nanoparticles (Figure 5) than PANI/GSPEs.

AuNPs are excellent electrical conductors; their incorporation in the polymer generated multiple active sites, which facilitated the electron transfer across the PANI matrix during the electrochemical processes.

#### 3.2.2. Competitive Assay

In order to develop the competitive aptamer-based assay for profenofos detection, the thiolated DNA capture probe (oligo-SH) was immobilised on modified graphite screen-printed working electrodes, and the hybridisation reaction with the concentration of biotinylated aptamer sequence (apt-BIO) was performed in accordance with previously reported studies [7,14].

**Figure 6:**
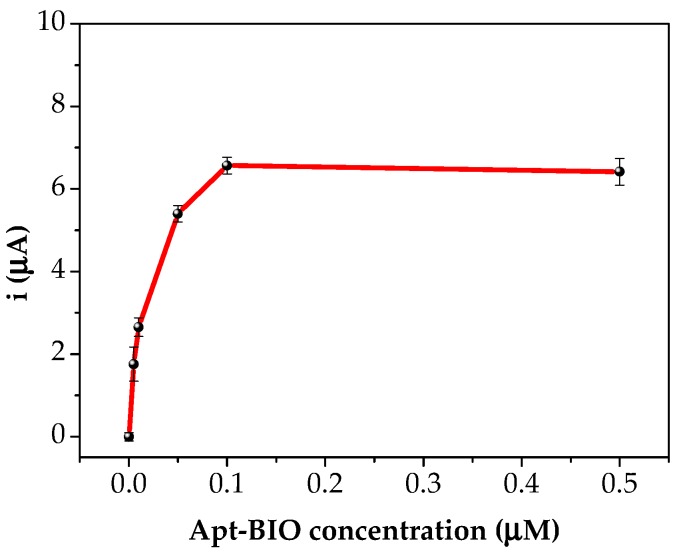
Hybridisation curve between immobilised oligonucleotide sequence (2.0 µM) and biotinylated aptamer solution in 0.50 M phosphate buffer, pH 7.0. Each point was repeated at least five times using different aptasensors.

Apt-BIO is functionalised with biotin in 5′ end, in order to interact with streptavidin-alkaline phosphatase enzyme conjugate. The current peak increased linearly with apt-BIO concentration up to 0.50 μM, where a plateau was reached (Figure 6).

This behaviour is due to the limited number of biorecognition sites that were bound onto the sensor surfaces. The concentration of 0.50 μM apt-BIO was thus used for all experiments involving profenofos, since to perform the competitive assay, it is necessary to work in saturation conditions to obtain the maximum signal value in absence of the analyte.

#### 3.2.3. Profenofos Detection

The pesticide detection was performed by competitive assay. In order to check if the pesticide itself, as an organophosphorus compound, could constitute an inhibition element for the phosphatase enzyme used in the labelling step, spectrophotometric measurements were carried out. Absorbance at 405 nm was collected for biotinylated aptamer sequence, profenofos and enzyme in the presence of *p*-nitrophenyl-phosphate enzymatic substrate. No significantly different values were obtained compared to the ones collected without profenofos, confirming that the pesticide is not interfering with the enzymatic activity (data not shown).

**Figure 7:**
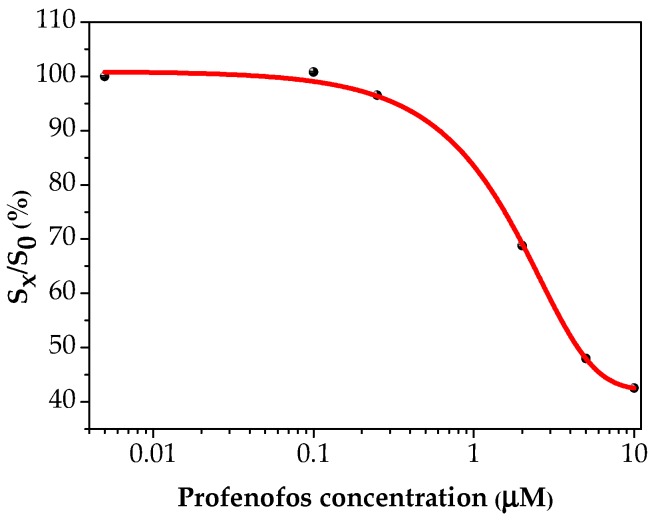
Profenofos dose-response curve. Each point was repeated at least 5 times using different aptasensors. The points correspond to % S_x_/S_o_ ± R.S.D. (error bars are smaller than data points).

Various profenofos concentrations containing 0.50 μM apt-BIO were thus evaluated. A dose-response curve of profenofos was obtained. The signal is reported as S_x_/S_0_ percent units, that is the percentage of the signal decrease with respect to the blank value. A decrement of the current peak height was recorded by increasing the concentration of the pesticide in the range 0.10–10 μM at a fixed concentration of the aptamer sequence (apt-BIO) (Figure 7).

The fitting of experimental data values gives the equation

(2) $$y=42.2+\frac{127.2}{1+e^{\left(x+0.3\right)/1.7}}$$

A correlation coefficient R^2^ of 0.997 was found. The detection limit (DL) was calculated as previously reported by Taylor et al. [23], and a value of 0.27 μM was obtained. The reproducibility of the aptasensor was also evaluated by multiple analysis of each standard profenofos solution.

Aptamer cross-reactivity of the proposed aptasensor was investigated in the presence of paraoxon, another OP pesticide. Paraoxon is the active metabolite of parathion, a molecule with a strong insecticidal and acaricidal effect, and the exposure to this molecule produces high mortality [24]. A value of 10% S_x_/S_0_ was observed when 1.0 μM of parathion solution was tested. The described aptasensor was confirmed as a promising analytical tool for pesticide detection, since the tested concentrations gave an average 0.2% RSD value for ten repetitions of each standard solution.

#### 3.2.4. Fruit Juice Samples Analysis

**Figure 8:**
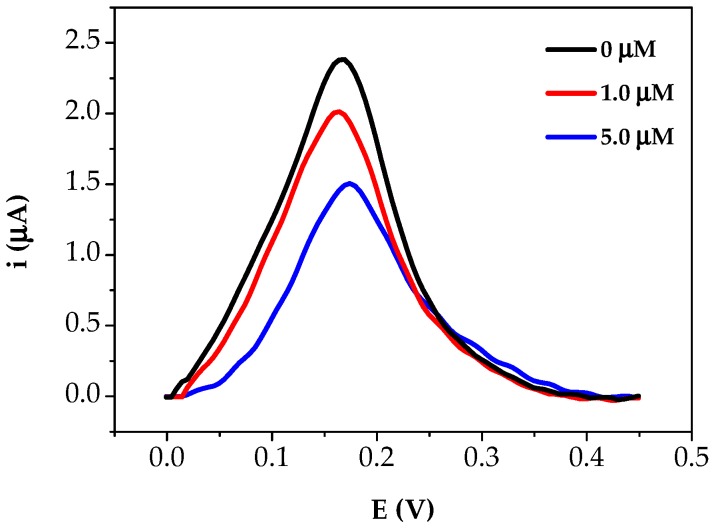
DPV scans of the analysed fruit juice samples spiked with profenofos solution.

In order to evaluate the practicability of the aptasensor, the preliminary experiments were performed in commercially available pear juice samples were carried out. Profenofos standard solutions were added to the samples after dilution (1:10) in phosphate buffer. The aptasensor response was then determined by DPV measurements, in the same conditions used for the pesticide calibration curve (Figure 8).

The signal was found to decrease with respect to the sample solution by increasing the profenofos spiked concentration in the analysed samples. The profenofos concentration in the pear juice samples was calculated through the obtained calibration curve. A little matrix effect was observed resulting the recovery value. The results obtained for the spiked samples with standard addition of profenofos are shown in Table 2.

## 4. Conclusions

Affinity-based biosensing can contribute to pesticide detection as a valid and innovative analytical approach. In this work, we developed, for the first time, a simple and cost-effective aptasensor for the direct determination of profenofos pesticide based on a competitive format and disposable screen-printed electrochemical cells. Preliminary experiments were performed with real samples. A dose response curve was obtained between 0.10 μM and 10 μM with a detection limit of 0.27 μM. The sensitivity achieved was adequate for the analysis of profenofos in real samples, although the highest level of a pesticide residue that is legally tolerated in or on food depends on the food nature. From the obtained results, this analytical tool has proven itself to be promising for application in real samples analysis, since it involved low amounts of reagents and easy-to-prepare portable aptasensors. All these features make it suitable for the realisation of a commercial kit. This allows in situ analysis that with traditional techniques such as chromatography is not achievable.

## Figures and Tables

**Table 1:** Melting temperature (T_m_) values, as obtained from analysing melting curves. The melting curves were performed with 1.0 μM biotinylated DNA aptamer (Apt-BIO), 1.0 μM thiolated DNA complementary (oligo-SH) and 1.0 μM profenofos solutions. The temperature was increased at a constant rate of 1.0 °C/min from 25 °C to 95 °C. Further details are described in the Section 2.3.

Sample	T_m_ (°C)
Apt-BIO	54.0
Apt-BIO + oligo-SH	58.0
Apt-BIO + profenofos	57.0

**Table 2:** Recoveries of pear juice samples spiked with profenofos standard solutions. Each concentration was repeated at least five times using different aptasensors.

Profenofos Spiked (µM)	Profenofos Found (µM)	Recovery (%)	Bias (%)	% RSD
1.0	0.87	87	−13	10
3.0	2.45	82	−18	15
